# Supplementary material for: What is associated with reported acute respiratory infection in children under 5 and PCV vaccination in children aged 1–36 months in Malawi? A secondary data analysis using the Malawi 2014 MICS survey
Source: PLoS One. 2023 Mar 31;18(3):e0283760. doi: 10.1371/journal.pone.0283760 (PMC10065275; doi:10.1371/journal.pone.0283760)
Supplement: S1 File — (DOCX) [file pone.0283760.s001.docx]

**What predicts reported acute respiratory infection in children under 5 and PCV vaccination in children aged 1-36 months in Malawi? A secondary data analysis using the Malawi 2014 MICS survey**

**Justine Gosling and Tim Colbourn**

**Supplementary Material:**

**Fig S1. Sample eligibility**

19,285 children aged under five in the children’s data base

Ineligible:

264 Not at home for interview

15 Refused

6 Partially completed

5 incapacitated

14 “Other” reason for incomplete interview

18,981 children aged under five available for analysis

**Fig S2 presenting the RARI sample in the 2 week period prior to the survey:**

Has your child had difficulty breathing during illness with cough (RARI) in the last 2 weeks?

n = 7,808

Missing n = 11,173

No

N = 3,426

(43.8%)

Yes

n = 4,382

(56.2%)

**Table S1** **Socio-economic associations with RARI in children under 5 including missing data on RARI**

| **Exposure variable** | **Measure** | **Chi squared uni variable analysis:**  **RARI** | | | **Chi Squared Uni- analysis**  **(95% CI)** |
| --- | --- | --- | --- | --- | --- |
|  |  | **Yes** | **No** | **Missing** |  |
| **Sex** | **Male**  **n = 9,490** | n = 2,218 (22.4%) | n = 1,713 (18.1%) | n=5,559  (58.6%) | p= 0.626 |
|  | **Female**  **n = 9,491** | n = 2,164 (22.8%) | n = 1,713 (18.1%) | n=5,614  (59.2%) |  |
|  | **Total**  **n = 18,981** | n = 4,382 (23.1%) | n = 3,426 (18.1%) | n=11,173  (58.9%) |  |
| **Region** | **Northern**  **n = 3,320** | n = 825 (24.9%) | n = 661 (19.9%) | n=1,834  (55.2%) | p=0.000* |
|  | **Central**  **n = 6,451** | n = 1,350 (20.9%) | n = 1,264 (19.6%) | n=3,837  (59.5%) |  |
|  | **Southern**  **n = 9,210** | n = 2,207 (24.0%) | n = 1,501 (16.3%) | n=5,502  (59.7%) |  |
|  | **Total**  **n = 18,981** | n = 4,382 (23.1%) | n = 3,426 (18.1%) | n=11,173  (58.9%) |  |
| **Area** | **Urban**  **n = 2,125** | n = 397 (18.7%) | n = 419 (19.7%) | n=1,309  (61.6%) | p=0.000* |
|  | **Rural**  **n =16,856** | n = 3,985 (23.6%) | n = 3,007 (17.8%) | n=9,864  (58.5%) |  |
|  | **Total**  **n = 18,981** | n = 4,382 (23.1%) | n = 3,426 (18.1%) | n=11,173  (58.9%) |  |
| **Age** | **0-5 months n = 1,686** | n = 359 (21.3%) | n = 273 (16.2%) | n=1,054  (62.5%) | p= 0.000* |
|  | **6-11 months**  **n = 1,791** | n = 472 (26.4%) | n = 348 (19.4%) | n=971  (54.2%) |  |
|  | **12-23 months**  **n = 3,870** | n =998 (25.8%) | n = 703 (18.2%) | n=2,169  (56.1%) |  |
|  | **24-35 months**  **n = 3,795** | n = 869 (22.9%) | n = 721 (19.0%) | n=2,205  (58.1%) |  |
|  | **36-47 months**  **n = 4,099** | n = 918 (22.4%) | n = 721 17.6%) | n=2,460  (60.0%) |  |
|  | **48-59 months**  **n = 3,740** | n = 766 (20.5%) | n = 660 (17.7%) | n=2,314  (61.9%) |  |
|  | **Total**  **n = 18,981** | n = 4,382 (23.1%) | n = 3,426 (18.1%) | n=11,173  (58.9%) |  |
| **Mothers education level** | **None**  **n = 2,528** | n = 488 (19.3%) | n = 373 (14.8%) | n=1,667  (65.9%) | p=0.000* |
|  | **Primary**  **n = 13,330** | n = 3,204 (24.0%) | n = 2,420 (18.2%) | n=7,706  (57.8%) |  |
|  | **Secondary**  **n = 2,921** | n = 653  (22.4%) | n = 579 (19.8%) | n=1,689  (57.8%) |  |
|  | **Higher**  **n = 188** | n = 35 (18.6%) | n = 47 (25.0%) | n=106  (56.4%) |  |
|  | **Total**  **n = 18,981** | n = 4,382 (23.1%) | n = 3,426 (18.1%) | n=11,173  (58.9%) |  |
| **Wealth quintile** | **Poorest**  **n = 4,290** | n = 1,027 (23.9%) | n = 699 (16.3%) | n=2,564  (59.8%) | p=0.000* |
|  | **Second**  **n = 4,190** | n = 1,040 (24.8%) | n = 716 (17.1%) | n=2,434  (58.1%) |  |
|  | **Middle**  **n = 4,082** | n = 936 (22.9%) | n = 736 (18.0%) | n=2,410  (59.0%) |  |
|  | **Fourth**  **n = 3,538** | n = 804 (22.7%) | n = 676 (19.1%) | n=2,058  (58.2%) |  |
|  | **Richest**  **n = 2,881** | n = 575 (20.0%) | n = 599 (20.8%) | n=1,707  (59.3%) |  |
|  | **Total**  **n = 18,981** | n = 4,382 (23.1%) | n = 3,426 (18.1%) | n=11,173  (58.9%) |  |
| **WHO Weight for height Z score** | **>-2 Zscores (normal)**  **n = 18,269** | n = 4,213 (23.1%) | n = 3,307 (18.1%) | n=10,749  (58.8%) | p= 0.883 |
|  | **-3 to -2 Zscores (low)**  **n = 508** | n =119 (23.4%) | n = 87 (17.1%) | n=302  (59.5%) |  |
|  | **<3 Zscores (severely low)**  **n = 204** | n = 50 (24.5%) | n = 32 (15.7%) | n=122  (59.8%) |  |
|  | **Total**  **n = 18,981** | n = 4,382 (23.1%) | n = 3,426 (18.1%) | n=11,173  (58.9%) |  |
| **Number of people residing in house** | **<5**  **n = 10,023** | n = 2,364 (23.6%) | n = 1,840 (18.4%) | n=5,819  (58.1%) | p= 0.159 |
|  | **6-10**  **n = 8,507** | n = 1,917 (22.5%) | n = 1,513 (17.8%) | n=5,077  (59.7%) |  |
|  | **11-25**  **n = 451** | n = 101 (22.4%) | n = 73 (16.2%) | n=277  (61.4%) |  |
|  | **Total**  **n = 18,981** | n = 4,382 (23.1%) | n = 3,426 (18.1%) | n=11,173  (58.9%) |  |
| **Number of children under 5 residing in house** | **1-3**  **n = 18,840** | n = 4,351 (23.1%) | n = 3,405 (18.1%) | n=11,084  (58.8%) | p= 0.523 |
|  | **4-6**  **n = 141** | n = 31 (22.0%) | n = 21 (14.9%) | n=89  (63.1%) |  |
|  | **Total**  **n = 18,981** | n = 4,382 (23.1%) | n = 3,426 (18.1%) | n=11,173  (58.9%) |  |
| **Childs birth order** | **1^st^**  **n = 8,194** | n = 1,957 (23.9%) | n = 1,493 (18.2%) | n=4,744  (57.9%) | p= 0.055 |
|  | **2^nd^**  **n = 4,846** | n = 1,087 (22.4%) | n = 874 (18.0%) | n=2,885  (59.5%) |  |
|  | **3^rd^**  **n = 2,654** | n = 569 (21.4%) | n = 477 (18.0%) | n=1,608  (60.6%) |  |
|  | **4^th^**  **n = 1,570** | n = 348 (22.2%) | n = 288 (18.3%) | n=934  (59.5%) |  |
|  | **5^th^**  **n = 689** | n = 152 (22.1%) | n = 117 (17.0%) | n=420  (61.0%) |  |
|  | **6^th^**  **n = 298** | n = 85 (28.5%) | n = 38 (12.8%) | n=175  (58.7%) |  |
|  | **7^th^**  **n = 114** | n = 27 (23.7%) | n = 15 (13.2%) | n=72  (63.2%) |  |
|  | **8^th^**  **n = 34** | n = 5 (14.7%) | n = 8 (23.5%) | n=21  (61.8%) |  |
|  | **Total**  **n = 18,399** | n = 4,230 (23.0%) | n = 3,310 (18.0%) | n=10,859  (59.0%) |  |
| **Location of cooking activities** | **Separate kitchen room**  **n = 1,553** | n = 373 (24.0%) | n = 251 (16.2%) | n=929  (59.8%) | p= 0.015 |
|  | **Elsewhere in house**  **n = 883** | n = 236 (26.7%) | n = 159 (18.0%) | n=488  (55.3%) |  |
|  | **Separate building**  **n = 11,128** | n = 2,585 (23.2%) | n = 2,062 (18.5%) | n=6,481  (58.2%) |  |
|  | **Outdoors**  **n = 5,240** | n = 1,157 (22.1%) | n = 915 (17.5%) | n=3,168  (60.5%) |  |
|  | **Other**  **n = 18** | n = 4  (22%) | n = 4  (22%) | n=10  (55.6%) |  |
|  | **Total**  **n = 18,822** | n = 4,355 (23.1%) | n = 3,391 (18.0%) | n=11,076  (58.9%) |  |
| **Cooking fuel used** | **Electric**  **n = 153** | n = 25 (16.3%) | n = 35  (22.9%) | n=93  (60.8%) | p=0.001* |
|  | **Kerosene**  **n = 1** | Not enough data for analysis n = 1 | | |  |
|  | **Coal**  **n = 3** | Not enough data for analysis n = 3 | | |  |
|  | **Charcoal**  **n = 2,142** | n = 426 (19.9%) | n = 431 (20.1%) | n=1,285  (60.0%) |  |
|  | **Wood**  **n = 16,611** | n = 3,902 (23.5%) | n = 2,953 (17.8%) | n=9,756  (58.7%) |  |
|  | **Straw/**  **shrubs**  **n = 46** | n = 20 (43%) | n = 5  (11%) | n=21  (46%) |  |
|  | **Crops**  **n = 18** | n = 6  (33%) | n = 2  (11%) | n=10  (56%) |  |
|  | **Other n = 1** | Not enough data for analysis n = 1 | | |  |
|  | **Total**  **n = 18,975** | n = 4,380 (23.1%) | n = 3,426 (18.1%) | n=11,169  (58.9%) |  |

*Indicates significance at the p <0.005 level

**Table S2 Children aged 1-36 months who have received the PCV vaccine since its introduction in November 2011 to the end survey date of April 2014, as evidenced from their record card:**

| **PCV vaccine dose** | **Number of children Under 5 (n)** |
| --- | --- |
| PCV1  n = 8,449 | Yes n = 7,761 (91.9%)  No n = 688 (8.1%) |
| PCV2  n = 8,426 | Yes n = 7,260 (86.2%)  No n = 1,166 (13.8%) |
| PCV3  n = 8,362 | Yes n = 6,529 (78.1%)  No n = 1,833 (21.9%) |

**Table S3 Vaccination report card and recall responses:**

| **Variable** | **Measure** | **Number of children Under 5** | |
| --- | --- | --- | --- |
| Asked: Has a vaccination card?  n = 11,052 | Excluded as not aged 1-36 months | n = 174 | |
|  | Yes - seen | n = 8,686 (78.6%) | |
|  | Yes – not seen | n = 1,195 (10.8%) | |
| Child ever given PCV vaccine if no vaccination card?  n = 2,122 | No | n = 1,171 (10.6%) | |
|  | Yes | n = 2,049 (94.9%) | |
|  | No | n = 109 (5.1%) | |
| If yes, how many given?  n = 2,005 | 1 | n = 200 (9.8%) | Accumulative total (assuming all those who had received PCV 3 also had 1 and 2)  n = 2,036 |
|  | 2 | n = 349 (17.1%) | Accumulative total (assuming all those who had received PCV 2 also had PCV1)  n = 1,836 |
|  | 3 | n = 1,487 (73.0%) | Accumulative total (assuming all those who had received PCV 3 also had PCV1 and 2)  n = 1,487 |
|  | Missing data | n = 13 | |

**Fig S3 showing composition of the PCVtotals variables:**

**PVC1total variable**

(All that received PCV1)

**n = 7,761 + 2,036 = 9,797**

PCV1 received on record card Yes n = 7,761 No n = 688

**PVC1total3**

(All that received PCV3)

**n = 6,529 + 1,487 = 8,016**

**PVC2total variable**

(All that received PCV2)

**n = 7,260 + 1,836 = 9,096**

PCV3 received on record card Yes n = 6,529 No n = 1,922

PCV2 received on record card Yes n = 7,262

No n = 1,255
