# Supplementary material for: What is associated with reported acute respiratory infection in children under 5 and PCV vaccination in children aged 1–36 months in Malawi? A secondary data analysis using the Malawi 2014 MICS survey
Source: PLoS One. 2023 Mar 31;18(3):e0283760. doi: 10.1371/journal.pone.0283760 (PMC10065275; doi:10.1371/journal.pone.0283760)
Supplement: Attachment — Submitted filename: PLOS ONE Review ARI MICs PCV Vax Malawi 06JUne2022.pdf [file pone.0283760.s004.pdf]

Peer Review

Journal: PLOS ONE

Number: PONE-D-21-21665

Title: What predicts reported acute respiratory infection in children under 5 and PCV vaccination in children aged 1-36 months in Malawi? A secondary data analysis using the Malawi 2014 MICS survey.

Date: June 06th, 2022

Peer Reviewer: Dr. Ezekiel Mupere MBChB, M.Med, MS., PhD (Epidemiology & Biostatistics)

Review Comments

Focus: Research – Secondary Data Analysis Using MICS Malawi

Originality: The research findings for this manuscript are relevant in contributing to the body of knowledge in understanding at national level, drivers of acute respiratory infection (ARI) and pneumococcal conjugate vaccine (PCV) uptake.

Importance and science: The findings in this manuscript are of public health importance. The authors set to study factors that predict PCV uptake and ARI in Malawi using MICS secondary data. The findings inform programmers on where to provide targeted interventions to improve PCV uptake or avert ARI in the country. The authors took effort to provide adequate background information about the current burden of ARI and PCV coverage, and how PCV vaccinations contribute to ARI reduction. Appropriate methods as regards study design, setting, sampling procedure, and measurements for how the primary data collection was conducted. The operational outcome definitions, explanatory variables and analytic approach were articulated.

However, several additional details may need to be addressed to improve clarity and interpretation of the findings for the manuscript:

Methods:

1. The study design for the secondary analysis was not described. The authors appear to have conducted a cross-sectional analysis of secondary. This may need to be articulated.
2. Selection criteria for the secondary analysis was not articulated. The details with explicit inclusion and exclusion criteria should be articulated.
3. Power calculations or computations for the planned study outcomes in secondary analysis – drivers of PCV uptake and ARI were not articulated to enable readers understand the strength or limitations that were inherent in detecting the anticipated differences.
4. Any possible misclassifications of the study outcomes and how this might have affected the measures of associations.
5. In the analytic strategy, it was important for the authors to have stated whether they explored for interaction or effect modification for certain factors on to the outcome. For example, region or area variable interacting with wealth index or other factors.

Results**:** The results are relevant to the study objective posed. Synthesis of results was satisfactory. However,

1. Presentation may need to be improved for Tables 1-5. Hereafter is draft example to improve the lay-out and use footnotes to any missing observations.

Table 1: Characteristics and Analytic Variables

**Variable**	**Measure**	**Frequency (n= 18,981)**
		n (%)
[1]Sex		
	Male	9,490 (50%)
	Female	9,491 (50%)

[1]Sex = 0, missing observations.

2. The authors needed to have explored interaction and presented results if interactions were present or significant say between region and wealth index on to ARI. If any of the interactions were found significant then then authors should present stratified analysis.

<u>Interpretation and conclusions:</u> The authors articulated the explanations for the claims and related with studies/findings from other settings or populations. However, authors may need to revise the discussion and consider the following:

1. Articulate study strengths or uniqueness of study findings with possible rebuttal.
2. Consider results that would arise when interactions are explored.
3. Discuss the limits of the external validity or generalizability of the findings.
4. Implications of study findings to further programs.

<u>References</u>: The references were relevant.

<u>Other points:</u>

1. The writing style was appropriate
