# Supplementary material for: What is associated with reported acute respiratory infection in children under 5 and PCV vaccination in children aged 1–36 months in Malawi? A secondary data analysis using the Malawi 2014 MICS survey
Source: PLoS One. 2023 Mar 31;18(3):e0283760. doi: 10.1371/journal.pone.0283760 (PMC10065275; doi:10.1371/journal.pone.0283760)
Supplement: Attachment — Submitted filename: Response to Reviewers (Gosling Malawi ARI PLOS ONE).docx [file pone.0283760.s005.docx]

Here we respond first to the journal requirements and then to each of the two reviewers –thanks very much for your reviews. Our responses are in blue.

**Journal Requirements:**

When submitting your revision, we need you to address these additional requirements.

1. Please ensure that your manuscript meets PLOS ONE's style requirements, including those for file naming. The PLOS ONE style templates can be found at 
<https://journals.plos.org/plosone/s/file?id=wjVg/PLOSOne_formatting_sample_main_body.pdf> and 
<https://journals.plos.org/plosone/s/file?id=ba62/PLOSOne_formatting_sample_title_authors_affiliations.pdf>

Authors response: we have ensured our manuscript meets PLOS ONE’s style requirements including those for file naming

2. Please provide further information in the data availability statement regarding the source of the 2014 UNICEF Malawi MICS data (for example, where the data were obtained and if any permission was required to use) as per our data availability policy (<https://journals.plos.org/plosone/s/data-availability#loc-acceptable-data-access-restrictions>).

Authors response: Thanks, we have clarified that the data are freely accessible by adding the following: “Anyone can access datasets, however, researchers need to register on the MICS website to gain access”

3. In your Data Availability statement, you have not specified where the minimal data set underlying the results described in your manuscript can be found. PLOS defines a study's minimal data set as the underlying data used to reach the conclusions drawn in the manuscript and any additional data required to replicate the reported study findings in their entirety. All PLOS journals require that the minimal data set be made fully available. For more information about our data policy, please see <http://journals.plos.org/plosone/s/data-availability>.

Upon re-submitting your revised manuscript, please upload your study’s minimal underlying data set as either Supporting Information files or to a stable, public repository and include the relevant URLs, DOIs, or accession numbers within your revised cover letter. For a list of acceptable repositories, please see <http://journals.plos.org/plosone/s/data-availability#loc-recommended-repositories>. Any potentially identifying patient information must be fully anonymized.

Important: If there are ethical or legal restrictions to sharing your data publicly, please explain these restrictions in detail. Please see our guidelines for more information on what we consider unacceptable restrictions to publicly sharing data: <http://journals.plos.org/plosone/s/data-availability#loc-unacceptable-data-access-restrictions>. Note that it is not acceptable for the authors to be the sole named individuals responsible for ensuring data access.

We will update your Data Availability statement to reflect the information you provide in your cover letter.

Authors response: We have uploaded the minimal data set, and the code (Stata .do file) required to reproduce the results in the paper. The first part of the code explains how the minimal data set was produced from the main 2014 UNICEF Malawi MICS data. The second part of the code produces the Tables in the paper. During the process of going through this we noticed a minor errors in our reporting of the outputs of our statistical analyses and in our creation of the number of people in the house and number of children aged under 5 in the house variables. These are very minor and none of them affect our interpretation or conclusions. We would be very happy for the editor or reviewers to re-run our analyses using the provided minimal data set and code, which we have been careful to annotate well to explain exactly what we did and how the results of running the code are the results reported in our paper.

4. Please note that in order to use the direct billing option the corresponding author must be affiliated with the chosen institute. Please either amend your manuscript to change the affiliation or corresponding author, or email us at [plosone@plos.org](mailto:plosone@plos.org) with a request to remove this option.

Authors response: The corresponding author is affiliated with UCL (University College London) who are the chosen institute for direct billing given UCL’s agreement with PLOS ONE. I hope this is all in order though please let us know if not.

**Reviewers comments:**

**Reviewer #1: Major comments**
1. The manuscript seems to contain more than two separate research questions, or not conceptually connected to each other. As shown in the Title - “What predicts reported acute respiratory infection in children under 5” and PCV vaccination in children aged 1-36 months in Malawi, though the data source is the same, have separate objective, and also separately analyzed and discussed. Thus, the manuscript has too much information and difficult to follow/understand
2. Authors indicated nearly 60% non-response on RARI (In the result section). With such a huge non-response rate, the validity of the research finding, and hence the conclusion of the RARI and also the national representativeness of the research is highly questioned.

Authors response: Thanks very much for your review of our paper. Both research questions are related to childhood respiratory infection and therefore relevant to cover in the same paper. We have tried to reduce the amount of information in the paper as per below, whilst also endeavouring to respond to requests from both peer reviewers of the paper.

With regards to your concerns on the nearly 60% non-response rate on RARI – throughout the paper we explain our justifications for its use and acknowledge the limitations of this measure, accepting that there is no optimal alternative and that RARI is widely used in the literature. Use of the term is also recommended by UNICEF and is relevant to health policy makers.

General comments

It is difficult to comment the manuscript at its current stage, as the manuscript contains chunk of information. But, generally the authors need to consider
1. The introduction section contains too much information which is not relevant for the paper/ lacks focusing on the objective. In addition, it has issues related with consistency/flow of information. The problem is not clear. Argument starts around “full vaccination” and “PCV’ going down the line.

Authors response: We have cut down the introduction considerably (please see tracked changes) including removing the second and third paragraphs containing background information about pneumonia, and Malawi, respectively, to focus only on the aspects relevant to our objectives. Our introduction is now only 786 words long.

3. Objective: Not usual to have four objectives (two primary and two secondary/specific) in scientific research papers. Hypotheses/ null hypothesis not clearly linked with the objectives

Authors response: Thanks for indicating your concern about the number of objectives we cover in our paper. We do not see a scientific problem here and would like to keep all 4 objectives. The hypotheses we state in our methods are clearly linked to our two primary objectives.

4. Methods: Too much explanation on the method used by UNICEF (MICS). Only the analysis section is inherent to this paper.

Authors response: We believe the explanation of the methods used by UNICEF (MICS) that we have briefly summarised in this paper are relevant to readers understanding of the data we used and would like to keep this information in the paper.

5. Results: The finding that, Over 56% of caregivers RARI in their children in the 2-week interview recall period, is very exaggerated because only the caregivers who respond to the question were included in the denominator.

Authors response: Thanks for highlighting this. We recognise that caregiver response to the question on reported acute respiratory infection (RARI) in the previous two-weeks may be subject to some selection bias and this is already included as a limitation of our paper, as explained in the second paragraph of the RARI sub-section in our Discussion:

“Survey data has been found to produce over estimates which may explain why our retrospective 2 week prevalence percentage is so high. In this survey, RARI was identified on the basis of caregivers reporting 2 symptomatic indicators; a cough and fast or difficulty in breathing, which were not clearly defined or quantified, potentially causing high levels of measurement bias. Interviewers relied on the inherent limited ability of caregivers to correctly recognise and RARI. Prevalence estimates from this survey should be interpreted with caution as they are based on caregivers perception of symptoms and their capacity to recall events up to 2 weeks later, which may be prone to recall bias. Another consideration is that the ARI indicator does not record severity of symptoms or illness specificity, e.g. whether symptoms are due to asthma, infection or pneumonia or whether symptoms just need antibiotics or are life threatening. Acknowledging this, the MICS refers to ARI rather than pneumonia and this is why pneumonia estimates composed from survey data, such as the MICS, are thought to greatly inflate the number of pneumonia cases with most reported episodes being false positives (Campbell et al, 2008).”

The discussion and conclusion need to be reviewed based on the above, if the authors accept the comments.

Authors response: Thanks, we have made the specific changes noted above and have also updated our conclusion to reflect this – the revised second sentence of our conclusion (see tracked changes) now reads:

“Whilst acknowledging the limitations of using survey data and the potential for recall bias, our cross-sectional analysis of secondary data reported social economic predictors for RARI in children under 5 and PCV vaccine uptake for children aged 1-36 months.”

**Reviewer 2**

Reviewer #2: The manuscript is of public health importance and was well written. However, major revisions have been suggested as regards study methods, analytic approach, results presentation and interpretations for consideration.

Methodology:

1. The study design for the secondary analysis was not described. The authors appear to have conducted a cross-sectional analysis of secondary. This may need to be articulated.

Authors response: Thanks very much for your review of our paper. We have added that our paper is a “cross-sectional analysis of secondary data” to our abstract, methods and conclusion (please see tracked changes).

2. Selection criteria for the secondary analysis was not articulated. The details with explicit inclusion and exclusion criteria should be articulated.

Authors response: Thanks, we have now clarified, at the beginning of our independent variables subsection of our methods (please see tracked changes) that: “We selected variables widely used in previous studies (see introduction) that aligned with our objectives”

3. Power calculations or computations for the planned study outcomes in secondary analysis – drivers of PCV uptake and ARI were not articulated to enable readers understand the strength or limitations that were inherent in detecting the anticipated differences.

Authors response: The statistical power of our analyses is apparent in the precision of the statistical results presented in our paper – the width of the confidence intervals, and corresponding p-values. We do not believe it is necessary or appropriate to show power calculations for secondary analyses of survey data, especially survey data like the MICS data we use, that was intended to be nationally representative and to have sufficient precision to explore the kinds of questions we answer in our paper. Power calculations are usually only appropriate in the planning stages of trials or intervention studies, and even then don’t need to be reported in trial results manuscripts when the precision of the actual results achieved is known and reported in the paper.

4. Any possible misclassifications of the study outcomes and how this might have affected the measures of associations

Authors response: We acknowledge the survey data is self-reported and that RARI is therefore likely to be much higher than true pneumonia – please see text in our Discussion on page 46:

“Prevalence estimates from this survey should be interpreted with caution as they are based on caregivers perception of symptoms and their capacity to recall events up to 2 weeks later, which may be prone to recall bias. Another consideration is that the ARI indicator does not record severity of symptoms or illness specificity, e.g. whether symptoms are due to asthma, infection or pneumonia or whether symptoms just need antibiotics or are life threatening. Acknowledging this, the MICS refers to ARI rather than pneumonia and this is why pneumonia estimates composed from survey data, such as the MICS, are thought to greatly inflate the number of pneumonia cases with most reported episodes being false positives (Campbell et al, 2008).

Despite the flaws of RARI and assumed over estimation of pneumonia cases in survey data, the RARI indicator in MICS surveys is still deemed valid as it is a quick and simple measure applicable in the field (Campbell et al, 2008). Given the prevalence and high mortality rates from pneumonia, there is an urgent need for research to measure the sensitivity and specificity of RARI for the identification of true pneumonia cases. It has been proposed that it is possible to increase test specificity without negatively impacting the study design by adding a few additional symptoms or signs to survey questionnaires and employing a “pneumonia score” (Hazir et, 2013). An evaluation of this measure is yet to be validated.”

Campbell, H., Biloglav, Z., Rudan, I. 2008. Reducing bias from test misclassification in burden of disease studies: use of test to actual positive ratio—new test parameter. *Croat Med J,* 49, pp. 402–414.

Hazir, T., Bequem, K., El Arifeen, S., Khan, A.M., Huque, M.H., et al. 2013. Measuring coverage in MNCH: A prospective validation study in Pakistan and Bangladesh on measuring correct treatment of childhood pneumonia. *PLoS Med* 10, (5).

5. In the analytic strategy, it was important for the authors to have stated whether they explored for interaction or effect modification for certain factors on to the outcome. For example, region or area variable interacting with wealth index or other factors.

Authors response: We did not explore potential effect modification for 3 reasons: 1) to limit the complexity of our analyses and enable clear interpretation of findings that can be more easily considered in the design of future policies and programmes, 2) some categories in our categorical variables are small meaning uncertain results (wide confidence intervals) and limited power to detect true interactions between variables, and 3) we do not expect significant effect modification between any of the variables included in our analyses. Wealth quintile is calculated from household assets in the same way for the whole of Malawi in the survey therefore should not be different by region.

Results:

1. Presentation may need to be improved for Tables 1-5. Hereafter is draft example to improve the lay-out and use footnotes to any missing observations.

Authors response: We note from the example table you sent that the only modification would be to remove “missing data” rows and include that information instead as footnotes. Our opinion is that this will not improve our Tables so we elect to leave them as they are and defer to the journal editor. We also note that the Tables will be more nicely typeset if the paper were to be published.

2. The authors needed to have explored interaction and presented results if interactions were present or significant say between region and wealth index on to ARI. If any of the interactions were found significant then then authors should present stratified analysis.

Authors response: Please see our response to point 5. above.

Interpretations and conclusions:

1. Articulate study strengths or uniqueness of study findings with possible rebuttal.

Authors response: Thanks, we have revised the first paragraph of our Discussion to now read:

“The findings of this study are of public health importance as it is the first that we know of, to predict nationally representative socio-economic factors for RARI in CU5 and PCV uptake in children aged 1-36 months in Malawi where the RARI burden is among the world’s highest. Our study also estimated vaccine coverage in 2014 for 1-36 year olds and calculated the average age a child received PCV doses in 2011/12/13/14. Our study can inform programmers on where to provide targeted interventions to improve PCV uptake or avert ARI in the country.

The strengths of the study are its large sample size and that we used publicly available UNICEF MICS data, therefore, this study could be easily repeated by others when the next survey is complete, to reliably monitor progress.”

2. Consider results that would arise when interactions are explored.

Authors response: Please see our response to point 5. above.

3. Discuss the limits of the external validity or generalizability of the findings.

Authors response: We believe we do discuss generalisability already in several places, and we acknowledge the limitations of the survey we use, from page 49:

“A major limitation of the survey is that it doesn’t account for significant confounding

comorbidities that predispose some children to pneumonia more than others, such

as HIV (Graham et al, 2011). Additionally, household surveys only include families

with a permanent residence and can exclude those most vulnerable with less access

to health care such as the homeless, displaced, children living in orphanages and

nomadic families (Carr-Hill 2012), thus possibly introducing selection bias.”

We also discuss the issues of measuring pneumonia via the ARI variable, and recall bias when vaccination cards aren’t available. Please also see our responses to Reviewer 1 above on these issues.

4. Implications of study findings to further programs:

Authors response: Thank you. We have now included this in our revised first paragraph of our Discussion.
