# Supplementary material for: What is associated with reported acute respiratory infection in children under 5 and PCV vaccination in children aged 1–36 months in Malawi? A secondary data analysis using the Malawi 2014 MICS survey
Source: PLoS One. 2023 Mar 31;18(3):e0283760. doi: 10.1371/journal.pone.0283760 (PMC10065275; doi:10.1371/journal.pone.0283760)
Supplement: Attachment — Submitted filename: Revision.docx [file pone.0283760.s006.docx]

The manuscript by Gosling et al. reports the results of a study conducted to identify the predictors of RARI in children under 5 and the predictors of PCV in children aged 1-36 months in Malawi.

The topic of the study is important and interesting, however, the objective of the study, the statistical models and the outcome definitions are poorly described and require additional elaboration.

In general, the topic of the study is important and interesting, however, the manuscript is difficult to read and there are some methodological issues to deal with. All the following comments do not exclude that the results of the analysis are correct and interesting, but represent suggestions to improve the paper and mostly his presentation.

In particular:

- **Abstract:** Authors should report the objectives of the study in the abstract.
- **Objectives:**
  - The definition of the study aims need to be revised. In particular, authors reported: “*1.The national, social economic predictors for RARI in children under 5; 2. The national, social economic predictors of PCV uptake in children aged 1-36 months. The 1-36 month age range reflects the available data from the MICS survey.*” I suggest to delete this last sentence (The 1-36 month age range reflects the available data from the MICS survey.) and to put it in the methods paragraph.
  - For what concern the secondary objectives, they are not really objectives but results that could be reported in the results paragraph as part of the sample description.
- **Methods:**
  - Could authors explain why they decided to consider children age as a predictor of PCV? I think that is difficult to interpret these results also because the dose of PCV (PCV1, PCV2, PCV3) depends on child age.
  - About the analysis of the data, I suggest to consider the possibility of putting together some variables categories that have not enough numerosity. For example, for *mother education*, authors could consider secondary and higher together; for *number of people residing in house* they can consider 11+ as a category; *child’s birth order* could be considered as linear and *cooking fuel used* could be recategorized. I this way the results would be more clears and stable.
  - In the **data analysis paragraph,** authors reported explanation about the chi squared test, the logistic regression and the p-value: “*A chi Squared analysis is appropriate for categorical data to statistically analyse frequency distribution (Sharpe 2015)*.”; “*Logistic regression analysis predicts the ratio of the odds of an event occurring, given the value of an independent variable compared to the reference category of the independent variable, e.g. the odds of RARI if rural living compared to urban living. Multiple logistic regression models examine the impact of multiple variables accounting for several potentially confounding variables simultaneously and is the appropriate regression analysis when the dependent variable is binary (Christiansen et al, 2015) as in this instance.*” “*A p value quantifies the significance of an association and the 95% CI quantifies the preciseness of the estimation with a values range (Kim and Bang, 2016) for which if the study was repeated multiple times, the true effect would be within this range 95% of the time (Dahiru 2008).”* I think that is not necessary to explain them in a scientific paper, what they should reported is how they use these methods, which are the dependent and independent variables, as they done in the previous paragraphs.
  - In the same way I suggest to delete the paragraphs related to hypotheses and null hypotheses, they are unnecessary if you have well described the objectives of your paper.
- **Results:**
  - **Tables** 2 and 6 are very difficult to read; if authors believe that is important to show all results and all steps of the analysis, I suggest to report a table with chi squared test with all variables and a table with the complete model, reporting crude and udjusted OR of the logistic regression analysis. Otherwise, I suggest to put the information about all tested variables that resulted not associated, only in the text or in additional material.
  - In the results paragraph (at page 36), authors reported: “*In all models, when compared to the 1-5 month age group, all age groups had statistically significant increased odds of PCV for all doses showing it to be an independent predictor of RARI, however the odds decreased with increasing age*”. Is not expected that the risk of being vaccinated increase with increasing age? Why authors do not analyze the risk of being vaccinated to PCV regardless to the number of doses?
  - I have some concern about the region (northern, central and southern) and the area (urban or rural); There are urban and rural areas in all regions or these variables are showing the same things?
  - In this paper authors took into account several predictors of RARI and PCV, some of these seem to represent similar aspects. Did authors evaluate collinearity or effect modification before establishing if these variables could be or not predictors of the outcomes?

MINOR COMMENTS:

- Tables titles need to be revised; for example, title of table 1 could be “Sample characteristics”; for table 2 (but also for others similar tables) I suggest to report something like “Socio economic predictors for RARI in children under 5 - results from the logistic regression model”.
- I do not understand as authors numbered figures in the paper. Figures 3 is the first figure of the paper, table 5 is after table 2.
