# Supplementary material for: What is associated with reported acute respiratory infection in children under 5 and PCV vaccination in children aged 1–36 months in Malawi? A secondary data analysis using the Malawi 2014 MICS survey
Source: PLoS One. 2023 Mar 31;18(3):e0283760. doi: 10.1371/journal.pone.0283760 (PMC10065275; doi:10.1371/journal.pone.0283760)
Supplement: Attachment — Submitted filename: Response to Reviewers Gosling Colbourn PLOS ONE R2.docx [file pone.0283760.s007.docx]

Thanks for your further careful reviews of our paper. We have further revised our paper accordingly. Please find our responses below each point in blue text.

**Reviewer #1:**

Dear authors/ editors

Thank you again for the opportunity to review the manuscript entitled "What predicts reported acute respiratory infection in children under 5 and PCV vaccination in children aged 1-36 months in Malawi? A secondary data analysis using the Malawi 2014 MICS survey"

I appreciate the authors for the improvements they made in the manuscript, particularly in the introduction section. Though, some editorial revisions are still needed. For instance

• Fig 3 is presented while Fig 1 and 2 are not available

Authors response: We have renumbered the Figures, and moved the figures and tables that were in the appendix at the end of the manuscript to a separate supplementary material document. The tables and figures in this supplementary material are now numbered Figure S1, S2, S3 and Table S1 and S2 as per PLOS One author guidelines.

• Zero missing values in table 1 are not important

Authors response: Given some variables are missing data in Table 1 we think it’s important to show readers that there is no missing data for the variables with no missing data.

• Ethical clearance is included under the data collection section, should be separated,

Authors response: Thanks, we have separated ethical approval into another sub-section titled “Ethical approval” below the “Data collection” sub-section of the methods.

• The selection of independent variables was referenced to (see introduction), but the introduction didn’t adequately address it.

Authors response: We believe the prior literature summarizing what is known about potential independent variables to include is summarized well in the introduction. Without explaining why you think it is not adequately addressed, we are unable to further improve it.

• Univariate analysis table shall be presented and discussed separately before the multivariate Table should

Authors response: this is an issue of presentation, and would involve additional tables and not presenting the univariable and multivariable results side by side, which we believe would detract from the clarity of the paper. We defer to the editor on this point.

• The numbers for the tables and figures are not correct and shall be edited

Authors response: As per our response to the first point above, we have corrected this and renumbered all the tables and figures in order in the text, and separately for the appendix, which we have now moved to a separate supplementary material document.

Major comments

I would like to re-emphasize on the previous comments, based on the authors response and in line with the developments in the manuscript

1. For the comment of having four objectives (two primary and two secondary/specific) and Hypotheses/ null hypothesis not clearly linked with the objectives, the authors respond that they do not see a scientific problem here and would like to keep all 4 objectives, and the hypotheses are clearly linked to our two primary objectives.

I agree that no problem of having more than one research question in a scientific paper. My intention is, for you to consider including very specific objectives under one or two comprehensive objectives, as the objectives are very similar. This would help to focus your discussion and presentation and also ease for readers.

Otherwise, authors shall consider addressing the statement of the problem and methods for each objective; and present and discuss each objective separately.

Authors response: thanks for your further consideration of this point. In response to related points from Reviewer 3 below, we have now simplified the paper by only stating the two primary objectives, and deleting the secondary objectives and hypothesis statements. We hope the paper is now clearer.

2.Methods section: Too much explanation on the method used by UNICEF (MICS). Only the analysis section is inherent to this paper.

Authors response: We believe the explanation of the methods used by UNICEF (MICS) that we have briefly summarized in this paper are relevant to readers understanding of the data we used and would like to keep this information in the paper.

Comment: I agree, but while a brief summary of the methods used by MICS is important, the methods used to for this secondary data study is more important for readers. It is important to inform readers, the design, how and what data has been extracted and organized, the operational definition, inclusion and exclusion criteria, etc., for the four objectives.

For instance the study population for RARI are U5C and for PCV coverage 1-36 months children are not the same and so is for data analysis etc., Thus, the authors shall address the methods (all subsections) used for each objective, as well as how the predictors of RARI and PCV uptake are selected

Authors response: thanks for your further consideration of this point. As per above, and in response to reviewer 3 we have simplified our paper to be focused on our two primary objectives. We believe we already describe how the data was extracted and organized, and why each of our variables were selected. We also include a clearly annotated Stata .do file as supplementary material, which clearly explains the steps of data cleaning, categorization of variables, and all of our analyses. Without details on specific issues remaining with any of these aspects, we are unable to further improve our manuscript.

Results: The finding that, Over 56% of caregivers RARI in their children in the 2-week interview recall period, is very exaggerated because only the caregivers who respond to the question was included in the denominator.

Authors response: Thanks for highlighting this. We recognize that caregiver response to the question on RARI in the previous two-weeks may be subject to some selection bias and this is already included as a limitation of our paper, as explained in the second paragraph of the RARI sub-section in our Discussion:

The previous comment is that the overestimation is created because the authors removed the participants who did not respond from the denominator, not due to selection bias or limitations inherent to survey methods.

The MICS 2014 reports 7.8% (~1475 of the 18981 children) with RARI (cough and fast/difficult breathing) were reported and that there was no non-response. Thus, how this study faced such a huge non-response rate, while using the same data? shall be explained and justified.

Authors response. Thank you for highlight this. From the reported two-week period prevalence of RARI in MICS (pages 55-57 of full MICS report and Table CH.4) it seems that the MICS report considers non-response (missing data in the dataset, as we tabulate in our paper) as “No” RARI. Table CH.4 in the MICS report does indeed show 7.8% of 18,981 children having “symptoms of ARI” but does not indicate for how many of the 18,981 children there is data on this indicator for. We show, via our replicable Stata .do file analyzing the publicly available MICS dataset that whilst 18,981 children have data on age and represent the overall denominator of children aged 0-59 months in the dataset (see lines 76-81 of our Stata .do file ‘Gosling_ARI_PLOS_ONE.do’; 18,981 is also the denominator in Table 1 in our paper), only 7,808 children have data on variable CA8 (“Difficulty breathing during illness with cough”, see questionnaire on page 637 of the MICS report), not 18,981. Of these 7,808, 56% (n=4,382) answer “Yes” to CA8. The full raw data on this variable from the raw MICS data (use minimal_dataset.dta and type “tab CA8 if CAGE!=., m” in Stata to tabulate CA8 for those not missing data on child age (to see overall 18.981) denominator, but displaying those missing data on CA8) is:

Difficulty	|

Breathing	|

during	|

illness	|

with cough	|	Freq.	Percent	Cum.

------------+----------------------------------------------------------------------

Yes	|	4,382	23.09	23.09

No	|	3,426	18.05	41.14

DK	|	4	0.02	41.16

Missing	|	10	0.05	41.21

.	|	11,159	58.79	100.00

------------+----------------------------------------------------------------------

Total |	18,981	100.00

This shows that 4 respondents answered “Don’t Know” (DK) and that 10 were indicated as “Missing” but that 11,159 children had no data on this variable at all in the dataset. This is not reported in the MICS report. Given the option for “Don’t Know” and “Missing”, as well as "Yes" and “No” we don’t believe it’s correct to assume that those without data are “No” (i.e. don’t have RARI). We have added the following text on page 20 of our paper to explain this:

“Of the 11,173 missing data on RARI (variable CA8 “Difficulty breathing during illness with cough” in the MICS dataset, see questionnaire on page 637 of MICS (2015)) only 4 responded “Don’t Know” and 10 were marked as “Missing” with the other 11,159 missing data on this variable in the dataset. We therefore don’t believe it’s correct to assume that those without data are “No” (i.e. don’t have RARI) and exclude them from our denominator.”

We do not know where the 7.8% “Symptoms of ARI” in Table CH.4 of the MICS report (which approximates to 1480 of 18,981 children) comes from as there are no other variables in the dataset apart from CA8 that pertain to “symptoms of ARI” (the only other variables related to this are CA7: “child ill with cough in last 2 weeks” – but this doesn’t include difficulty breathing; and CA9: “Symptoms due to problem in chest or blocked nose” – also not cough and difficulty breathing ARI definition, and neither of these have categories close to 1480 children)

In addition, in such huge nonresponse rate, it is important to analyze the non-responses against the independent variables and explain to readers the characteristics of those who are missing and how it may affect the result.

Authors response: thanks for this suggestion, we have now done this and added it as Table S1 in the supplementary material of our paper. This Table is the same as the first 5 columns of Table 2 of our paper, but with an added column for “missing data” on RARI, in addition to the “Yes” and “No” columns. From this, we can see that the proportion in each category of each variable missing data on RARI are similar – the proportion missing data on RARI only varies by 8 percentage points (e.g. for child age) or less across the categories of each socio-economic variable (Table S1). Therefore, our main RARI analysis (Table 2) is unlikely to be biased. We have added the following text on page 20 of our paper to explain this:

“We report socio-economic characteristics of those with missing data on RARI separately in supplementary Table S1 and find the proportion missing data on RARI only varies by 8 percentage points (e.g. for child age) or less across the categories of each socio-economic variable (Table S1), suggesting our analyses below are unlikely to be biased by this missing data.”

PCV: Similar to RARI, the number/percent of children 1-36 months enrolled in PCV up take, and non-response rate should be also presented before going to the analysis.

Authors response: Nearly all children aged 1-36 months in the dataset have data on whether they have a vaccine card so are part of our PCV analysis (Tables 3 and 4). Children aged 37-59 months are completely missing data on vaccination including PCV, but they are not part of this analysis as we’ve explained, now with added text to clarify, in our data collection section on page 10:

“Analysis of PCV can only be measured for 1-36 month olds as per available data from the MICS database and vaccine age criteria (there is no vaccination data on children aged 37-59 months). This age group permits children born after the introduction of the PCV in November 2011 up to the survey end date in April 2014.

In Stata, using minimal_dataset.dta run up to line 253, and see the output of line 253: tab CAGE IM1, m. Only 36 children aged 1-35 months don’t have data on whether they have a vaccine card (are not included in our Tables 3 and 4). An additional 200 children aged 36 months don’t have data on whether they have a vaccine card). We have added the following text to the beginning of our PCV Results section on page 33:

“Only 2% (n=236) of the 11,462 children aged 1-36 months did not have data on whether or not they had a vaccination card therefore we do not report non-response separately in our PCV analysis.”

PCV 3 coverage: The nationally representative study of children aged 1-36 months estimated that 77.0% of children had received all 3 PCV doses, less than the 86.0% estimated by Bondo et al., (2018) (PP 50). ?? Are the less than 3-month children expected to complete PCV 3 and included in the denominator? –

Authors response: We comment on the difference between our study and Bondo et al (2018) in detail in our discussion (page 52). We have just added one further clarification (in bold below, and in tracked changes in the paper) that, in addition to being in rural areas only, Bondo et al was limited to one district (Lilongwe):

“Our nationally representative study of children aged 1-36 months estimated that 77.0% of children had received all 3 PCV doses, less than the 86.0% estimated by Bondo et al., (2018) in rural children only, **in one area (Kabadula) of one district (Lilongwe) of Malawi,** aged between 6 weeks and 16 months. This could be explained by this studies findings in univariable logistic regression analysis that rural children have 0.82 reduced odds of receiving all 3 PCV vaccinations when compared to urban (p=0.003, CI 0.71-0.93, however in multivariable regression there was no difference found, perhaps because of confounding of rural dwelling with other explanatory variables e.g. lower education, lower wealth quintile. That rural children were found to have decreased odds of PCV vaccination in univariable analysis is unsurprising and has previously been explained by longer distances to travel for health services Munthali (2007).”

Less than 3 month old children are not expected to have completed PCV3 (that is due at 14 weeks as per the 6, 10, 14 week schedule) though, as our results show, the vaccine schedule was not always kept to (and some children younger than the schedule were also vaccinated according to the MICS data). We don’t adjust the denominator so as to keep the denominator consistent across the 3 groups for comparison between them. Given the vaccine schedule was not always followed, adjusting the denominators for each dose would be difficult to get right without excluding vaccinated children as well (which we don’t want to do), and it would only make a very small difference to the results.

**Reviewer #3**: The manuscript by Gosling et al. reports the results of a study conducted to identify the predictors of RARI in children under 5 and the predictors of PCV in children aged 1-36 months in Malawi.

The topic of the study is important and interesting, however, the objective of the study, the statistical models and the outcome definitions are poorly described and require additional elaboration.

In general, the topic of the study is important and interesting, however, the manuscript is difficult to read and there are some methodological issues to deal with. All the following comments do not exclude that the results of the analysis are correct and interesting, but represent suggestions to improve the paper and mostly his presentation.

In particular:

- **Abstract:** Authors should report the objectives of the study in the abstract.

Authors response: We believe our objectives were implicit following from the last sentence of our introduction in our abstract, though have now made them explicit, whilst adding only a few words in an additional sentence at the end of the introduction section of our abstract to keep the abstract concise. The last two sentences of the introduction section of our abstract now read:

“There has been no national study in Malawi that seeks to predict social economic factors leading to PCV vaccine uptake and reported acute respiratory infections (RARI). The objectives of our study were to do this.”

- **Objectives:**
  - The definition of the study aims need to be revised. In particular, authors reported: “*1.The national, social economic predictors for RARI in children under 5; 2. The national, social economic predictors of PCV uptake in children aged 1-36 months. The 1-36 month age range reflects the available data from the MICS survey.*” I suggest to delete this last sentence (The 1-36 month age range reflects the available data from the MICS survey.) and to put it in the methods paragraph.

Authors response: We have deleted this as recommended. We have emphasized it in the data collection section of our methods on page 10.

- For what concern the secondary objectives, they are not really objectives but results that could be reported in the results paragraph as part of the sample description.

Authors response: We have deleted the statement of our secondary objectives at the end of the introduction as recommended. We already report the results of these on pages 33-36.

- **Methods:**
  - Could authors explain why they decided to consider children age as a predictor of PCV? I think that is difficult to interpret these results also because the dose of PCV (PCV1, PCV2, PCV3) depends on child age.

Authors response: We included age to demonstrate that for many reasons, children may not get the vaccine at the recommended age, and this delay could have consequences for the child’s health and may explain some of our findings.

- About the analysis of the data, I suggest to consider the possibility of putting together some variables categories that have not enough numerosity. For example, for *mother education*, authors could consider secondary and higher together; for *number of people residing in house* they can consider 11+ as a category; *child’s birth order* could be considered as linear and *cooking fuel used* could be recategorized. I this way the results would be more clears and stable.

Authors response: Thank you for these suggestions, we have taken them on board and have made some changes to number of people residing in the house, and child’s birth order:

For number of people residing in the house: as there are only 21 observations in the 16-20 category, 3 in the 21-25 category, so we have done as you suggest and have combined 11-15, 16-20 and 21-25 into one category: 11-25. This does not alter our findings in our RARI analysis (Table 2), or our PCV analysis (Table 6) as this variable is not significant in univariable analyses, and is therefore also not in our multivariable analyses (so does not change them).

For child’s birth order (RARI analysis, Table 2) we have changed it to a linear variable in our logistic regression analyses. We agree this makes more sense given the isolated finding from the categorical variable of the 6^th^ born child being more at risk than the 7^th^, 8^th^ or others. We find no significant difference when child’s birth order is treated as a linear variable. We have updated our RARI multivariable analysis accordingly (child’s birth order is not included in our multivariable analysis now as it is not significant in our univariable analysis). As shown by our tracked changes the results for other variables in the multivariable analysis only change very slightly, and the significance of the results does not change.

For mothers education, there are 82 and 121 women with higher education in the RARI (Table 2) and PCV (Table 6) analyses respectively–sufficient numbers to include as a separate category, and higher education is quite different to secondary education so we have kept it as it is.

For cooking fuel used these are all very different and we don’t see a rational option for combining categories, so we choose to keep it as it is.

In the **data analysis paragraph,** authors reported explanation about the chi squared test, the logistic regression and the p-value: “*A chi Squared analysis is appropriate for categorical data to statistically analyse frequency distribution (Sharpe 2015)*.”; “*Logistic regression analysis predicts the ratio of the odds of an event occurring, given the value of an independent variable compared to the reference category of the independent variable, e.g. the odds of RARI if rural living compared to urban living. Multiple logistic regression models examine the impact of multiple variables accounting for several potentially confounding variables simultaneously and is the appropriate regression analysis when the dependent variable is binary (Christiansen et al, 2015) as in this instance.*” “*A p value quantifies the significance of an association and the 95% CI quantifies the preciseness of the estimation with a values range (Kim and Bang, 2016) for which if the study was repeated multiple times, the true effect would be within this range 95% of the time (Dahiru 2008).”* I think that is not necessary to explain them in a scientific paper, what they should reported is how they use these methods, which are the dependent and independent variables, as they done in the previous paragraphs.

Authors response: Thank you for this suggestion, we think the detail is useful though and shows complete transparency and thinking. We therefore would like to keep it in, unless the reviewer, or editor, feels strongly about it, in which case we can remove it.

- In the same way I suggest to delete the paragraphs related to hypotheses and null hypotheses, they are unnecessary if you have well described the objectives of your paper.

Authors response: Thank you for this suggestion, we have deleted the hypothesis statements.

- **Results:**
  - **Tables** 2 and 6 are very difficult to read; if authors believe that is important to show all results and all steps of the analysis, I suggest to report a table with chi squared test with all variables and a table with the complete model, reporting crude and udjusted OR of the logistic regression analysis. Otherwise, I suggest to put the information about all tested variables that resulted not associated, only in the text or in additional material.

Authors response: Thank you for this suggestion. We agree Table 2 and Table 6 (now numbered Table 4) are long and each spread over many pages currently. However, they will be much clearer when typeset for publication. We believe displaying all the results together is important to clearly demonstrate the difference between crude and adjusted analyses and to acknowledge all of the variables included. This is especially important for our multivariable logistic regression, to show all of the variables included in the same model.

- In the results paragraph (at page 36), authors reported: “*In all models, when compared to the 1-5 month age group, all age groups had statistically significant increased odds of PCV for all doses showing it to be an independent predictor of RARI, however the odds decreased with increasing age*”. Is not expected that the risk of being vaccinated increase with increasing age?

Authors response: Yes we expect this, but we want to quantify it, and also include age in our multivariable logistic regressions to adjust for it when considering the other variables in the models.

Why authors do not analyze the risk of being vaccinated to PCV regardless to the number of doses?

Authors response: We are interested in associations with each dose. The PCV1 results can be considered a proxy for any PCV given PCV1 is most common, and is required for subsequent doses (PCV2 and PCV3), i.e. PCV2 and PCV3 are subsets of PCV1.

- I have some concern about the region (northern, central and southern) and the area (urban or rural); There are urban and rural areas in all regions or these variables are showing the same things?

Authors response: These are separate variables. Malawi is divided into three regions: northern, central and southern. Like Malawi as a whole, each region is predominantly rural, though contains some urban areas. Therefore, seeing whether there are differences in our outcome variable by region, or by urban and rural areas are distinct things.

- In this paper authors took into account several predictors of RARI and PCV, some of these seem to represent similar aspects. Did authors evaluate collinearity or effect modification before establishing if these variables could be or not predictors of the outcomes?

Authors response: As per our above responses we believe all of the variables in our models are different and interesting in their own right, and have recategorized a couple as per your suggestions. We therefore have no theoretical reason to expect collinearity to be an issue (e.g. like birthweight and gestational age might be in an analysis of newborn outcomes). Given all of our models converge and there are no coefficients with extreme values or coefficients that could not be computed we do not believe there are any issues of collinearity in practice either.

As explained in our response to the previous Reviewer 2 in August, we did not explore potential effect modification for 3 reasons: 1) to limit the complexity of our analyses and enable clear interpretation of findings that can be more easily considered in the design of future policies and programmes, 2) some categories in our categorical variables are relatively small meaning wide confidence intervals and limited power to detect true interactions between variables, and 3) we do not expect significant effect modification between any of the variables included in our analyses.

MINOR COMMENTS:

- Tables titles need to be revised; for example, title of table 1 could be “Sample characteristics” for table 2 (but also for others similar tables) I suggest to report something like “Socio economic predictors for RARI in children under 5 - results from the logistic regression model”.

Authors response: Thanks for these suggestions, we have changed the titles of our tables accordingly. We prefer to keep the word “associations” rather than “predictors”.

- I do not understand as authors numbered figures in the paper. Figures 3 is the first figure of the paper, table 5 is after table 2.

Authors response: We have renumbered the Figures, and moved the figures and tables that were in the appendix at the end of the manuscript to a separate supplementary material document. The tables and figures in this supplementary material are now numbered Figure S1, S2, S3 and Table S1 and S2 as per PLOS One author guidelines, and the tables and figures in the paper are numbered sequentially in the order they are cited in the text.
