# Supplementary material for: What is associated with reported acute respiratory infection in children under 5 and PCV vaccination in children aged 1–36 months in Malawi? A secondary data analysis using the Malawi 2014 MICS survey
Source: PLoS One. 2023 Mar 31;18(3):e0283760. doi: 10.1371/journal.pone.0283760 (PMC10065275; doi:10.1371/journal.pone.0283760)
Supplement: Attachment — Submitted filename: review_PlOSone_rh.docx [file pone.0283760.s008.docx]

This paper used 2014 UNICEF Malawi Multiple Cluster Indicator Survey to analyse socio economic predictors for RARI un CU% and PCV uptake in children aged 1-36 months. I hope the authors find the following comments and suggestions helpful.

Major comments:

1. I would advise that the authors use the term "association" instead of "predictor" in the title and throughout the study, as "predictor" may mislead readers about the nature of the research conducted. In certain instances, the term "predictor" indicates to certain readers that causal analysis is performed. But in this paper's study, "endogeneity" is obviously overlooked. Therefore, it is more suitable to avoid the term "predictor" and instead use "association" in this context.
2. I would present a missing analysis, do a sensitivity analysis using imputed values for missing data, and report on how the analysis altered (if any) as a result.
3. Clearly the PCV uptake impacts the RARI in children. Thus, in Table 2 I would add the PCV uptake variable as an independent variable in the analysis. Otherwise this will create omitted variable bias.
4. The price type of the fuel used for cooking might be used as an indication of socioeconomic class. I would advise grouping them as expensive, moderately priced, and inexpensive cooking fuels and using that variable instead of the actual cooking fuel (electricity, coal, wood etc) used.
5. In PCV analysis it would be great and much more useful if a bivariate constabulary analysis of PCV uptake and socioeconomic indicators are reported.
6. The discussion part might be enhanced by arguing in accordance with the policy context and comparing the findings to those of other nations. This would be beneficial for international readers.

Minor comments:

1. Add CU5 abbreviation
