# Supplementary material for: What is associated with reported acute respiratory infection in children under 5 and PCV vaccination in children aged 1–36 months in Malawi? A secondary data analysis using the Malawi 2014 MICS survey
Source: PLoS One. 2023 Mar 31;18(3):e0283760. doi: 10.1371/journal.pone.0283760 (PMC10065275; doi:10.1371/journal.pone.0283760)
Supplement: Attachment — Submitted filename: Response to reviewer 5 Gosling ARI.docx [file pone.0283760.s009.docx]

Thanks for your additional review of our paper. We have further revised our paper accordingly. Please find our responses below each point in blue text.

Reviewer #5: This paper used 2014 UNICEF Malawi Multiple Cluster Indicator Survey to analyse socio economic predictors for RARI un CU% and PCV uptake in children aged 1-36 months. I hope the authors find the following comments and suggestions helpful.
Major comments:
1. I would advise that the authors use the term "association" instead of "predictor" in the title and throughout the study, as "predictor" may mislead readers about the nature of the research conducted. In certain instances, the term "predictor" indicates to certain readers that causal analysis is performed. But in this paper's study, "endogeneity" is obviously overlooked. Therefore, it is more suitable to avoid the term "predictor" and instead use "association" in this context.

Authors response: Thank you, we have changed “predictor” to “association” throughout.

2. I would present a missing analysis, do a sensitivity analysis using imputed values for missing data, and report on how the analysis altered (if any) as a result.

Authors response: Please see our response to concerns about missing data in our previous revision (R2), which included the newly added (in R2) supplementary Table S1:

Authors response: thanks for this suggestion, we have now done this and added it as Table S1 in the supplementary material of our paper. This Table is the same as the first 5 columns of Table 2 of our paper, but with an added column for “missing data” on RARI, in addition to the “Yes” and “No” columns. From this, we can see that the proportion in each category of each variable missing data on RARI are similar – the proportion missing data on RARI only varies by 8 percentage points (e.g. for child age) or less across the categories of each socio-economic variable (Table S1). Therefore, our main RARI analysis (Table 2) is unlikely to be biased. We have added the following text on page 20 of our paper to explain this:

“We report socio-economic characteristics of those with missing data on RARI separately in supplementary Table S1 and find the proportion missing data on RARI only varies by 8 percentage points (e.g. for child age) or less across the categories of each socio-economic variable (Table S1), suggesting our analyses below are unlikely to be biased by this missing data.”

This sensitivity analysis strongly suggests that imputing missing data would not significantly alter our results or conclusions.

3. Clearly the PCV uptake impacts the RARI in children. Thus, in Table 2 I would add the PCV uptake variable as an independent variable in the analysis. Otherwise this will create omitted variable bias.

Authors response: As we explain in our paper, the PCV data is only available for children aged 1-36 months. Our RARI analysis is separate and for all children aged under 5 years old (0-59 months) therefore we can’t include PCV in this analysis as it would change the sample for our RARI analysis completely.

4. The price type of the fuel used for cooking might be used as an indication of socioeconomic class. I would advise grouping them as expensive, moderately priced, and inexpensive cooking fuels and using that variable instead of the actual cooking fuel (electricity, coal, wood etc) used.

Authors response: There is no data on how much the fuel costs in the dataset (MICS 2014) that we use or on how much fuel each household uses so we are unable to determine the amount spent on fuel accurately. We also already include wealth quintile in our analysis separately.

5. In PCV analysis it would be great and much more useful if a bivariate constabulary analysis of PCV uptake and socioeconomic indicators are reported.

Authors response: In Table 4 we already report results separately for each dose of PCV: PCV1, PCV2, and PCV3 by socio-economic indicators both bivariate for each variable, and multivariable. We are not familiar with “constabulary analysis” and can’t find reference to it online.

6. The discussion part might be enhanced by arguing in accordance with the policy context and comparing the findings to those of other nations. This would be beneficial for international readers.

Authors response: This study uses nationally representative data from Malawi only and is therefore highly specific to the Malawian context. We therefore do not think it is appropriate to compare with policies of other countries, and the manuscript is already very long.


Minor comments:
1. Add CU5 abbreviation

Authors response: Thank you, we have added CU5 to our list of abbreviations, and added it in brackets after “children under 5” the first time it is mentioned in the abstract.
